# Synthesis and Evaluation of Some 17-Acetamidoandrostane and *N,N*-Dimethyl-7-deoxycholic Amide Derivatives as Cytotoxic Agents: Structure/Activity Studies

**DOI:** 10.3390/molecules18077436

**Published:** 2013-06-26

**Authors:** Yanmin Huang, Jianguo Cui, Linyi Jia, Chunfang Gan, Huacan Song, Chun Zeng, Aimin Zhou

**Affiliations:** 1 College of Chemistry and Life Science, Guangxi Teachers Education University, Nanning 530001, China; 2 No. 37 Middel School in Nanning, Nanning 530001, China; 3 School of Chemistry and Chemical Engineering, SUN YAT-SEN University, Guangzhou 510275, China; 4 Clinical Chemistry Program, Department of Chemistry, SI 424, Cleveland State University, Cleveland, OH 44115, USA

**Keywords:** pregnenolone, 17-acetamidoandrostane, *N,N*-dimethyl-7-deoxycholic amide, antiproliferative activity

## Abstract

Using pregnenolone and 7-deoxycholic acid as starting materials, some 17-acetamidoandrostane and *N,N*-dimethyl-7-deoxycholic amide derivatives were synthesized. The cytotoxicity of the synthesized compounds was tested *in vitro* against two tumor cell lines: SGC 7901 (human gastric carcinoma) and Bel 7404 (human liver carcinoma). The result showed that the blockage of the interaction of the amide group with outside groups might cause a decrease of the cytotoxicity, and an O-benzyloximino group at the 3-position of *N,N*-dimethyl-7-deoxycholic amide could enhance the cytotoxic activity of the compound. The information obtained from the studies provides the structure-activity relationship for these compounds and may be useful for the design of novel chemotherapeutic drugs.

## 1. Introduction

Steroidal compounds display a variety of biological functions and play a very important role in life [1,2,3]. Azahomosteroids are also a class of steroid compounds which were synthesized and modified in order to increase the biological activity of steroids. These compounds have been tested successfully as anti-cancer drugs against several types of cancer cells [4,5,6,7,8]. Some researchers indicated that the presence of the characteristic group (-NH-CO-) in the aza-homosteroid molecule had been proven to be important in lowering the acute toxicity and improving antitumour activity of the compound in cancer research [9,10].

**Figure 1:**
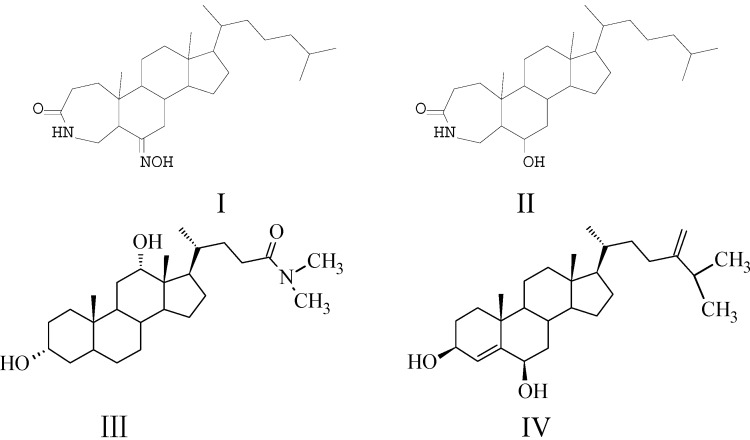
The structure of compounds I–IV.

Several new steroidal lactams with the introduction of NHCO groups on the A-ring, C-ring or D-ring of the steroidal nucleus were synthesized and assayed in our group [11,12,13,14]. We found that these compounds displayed distinct cytotoxicity against different cancer cells, for instance, the steroidal lactam compounds **I** and **II** (Figure 1) displayed distinct cytotoxicity against some cancer cells through effective induction of cancer cell apoptosis by activation of the intrinsic pathway [15].

**Scheme 1:**
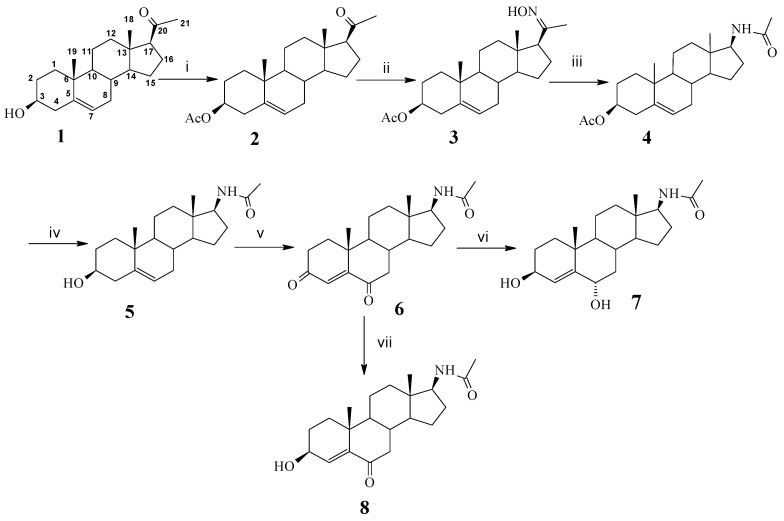
Synthetic procedures of 17-acetamido-androst-5-en derivatives.

**Scheme 2:**
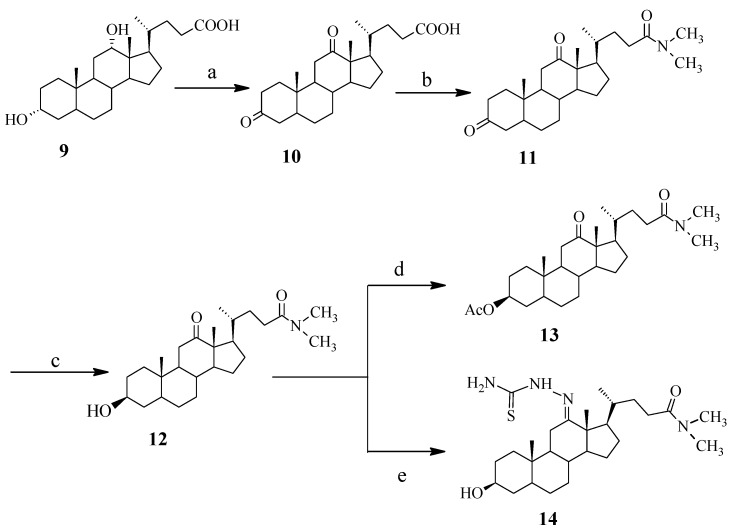
Synthetic procedures of *N,N*-dimethyl-7-deoxycholic amide derivatives.

A survey of the literature suggested steroidal compounds with a NHCO group in the side chain exhibit some valuable biological activities, such as cytotoxicity and antibacterial properties. For example, finasteride, dutasteride and PNU-157706 [16,17], used as clinical benign prostatic hyperplasia drugs, have a NHCO group in their branch chain. In order to further evaluate the antiproliferative activity of new steroidal derivatives and study their structure-activity relationships, some 17-acetamidoandrostane (Scheme 1) and N,N-dimethyl-7-deoxycholic amide derivatives (Scheme 2) with a NHCO group in their side chain were designed and synthesized. Their antiproliferative activities were tested *in vitro* against two tumor cell lines: SGC 7901 (human gastric carcinoma) and Bel 7404 (human liver carcinoma). 

Here, the *N,N*-dimethyl-7-deoxycholic amide (**III**) has a similar atom rank of the side chain of as 24-methylenecholest-4-en-3β,6β-diol (**IV**), which is a polyhydroxylsterol obtained from the soft coral *Alcyonium patagonicum* that showed potent cytotoxicity to murine leukemia cell with an IC_50_ value of 1 µg/mL [18], except for different atoms substituted in the 25- and 28-positions of the side chain.

*Reagents and conditions*: (i) Ac_2_O/Pyridine/r.t., 24 h; (ii) NH_2_OH.HCl/NaOAc.3H_2_O/95% CH_3_CH_2_OH, 60 °C, 2h; (iii) SOCl_2_/THF, r.t., 2 h; (iv) 13% K_2_CO_3_/CH_3_OH/reflux, 4 h; (v) Jones’ reagent/acetone/r.t., 2 h; (vi) NaBH_4_/CH_3_OH, r.t., 30 min; (vii) NiCl_2_·6H_2_O/NaBH_4_.

*Reagents and conditions*: (a) Jones’ reagent, (CH_3_)_2_CO, 0 °C; (b) Toluene, (CO)_2_Cl_2_, Py/(CH_3_)_2_NH (33%); (c) NaBH_4_/CH_3_OH; (d) Ac_2_O/Pyridine/r.t., 24 h; (e) NH_2_NHCSNH_2_/EtOH, 80 °C.2. Results and Discussion

## 2. Results and Discussion

### 2.1. Chemistry

Scheme 1 outlines the synthetic procedures for compounds **4**–**8**. First, the 3β-hydroxy group of pregnenolone (**1**) was protected by acetylation, then oximation of 3β-acetoxypregnenolone (**2**) was performed using NH_2_OH·HCl and NaOAc·3H_2_O to give compound **3**. Compound **4** was generated by Beckmann rearrangement of **3** with SOCl_2_/THF. Afterwards, compound **5** was obtained via the deacetylation of **4** with alcoholic K_2_CO_3_. Compound **6** was prepared by the oxidation of **5** with Jones’ reagent and multiple extraction because of better water solubility of compound **6**. Lastly, compound **6** was converted to compounds **7** and **8** by selective reduction using NaBH_4_ under different conditions.

Synthetic procedures to obtain *N*,*N*-dimethyl-7-deoxycholic amide derivatives **11**–**14** are outlined in Scheme 2. First, 3,12-dioxo-7-deoxycholic acid (**10**) was obtained by oxidation of 7-deoxycholic acid (**9**) with Jones’ reagent. Then, the reaction of **10** with (CO)_2_Cl_2_ and (CH_3_)_2_NH in toluene gave *N,N-*dimethyl-3,12-dioxo-7-deoxycholic amide (**11**). The structure of compound **11** had been confirmed by its IR, NMR and MS spectra. In the IR spectrum, the disappearance of the broad absorption peak at 3,200–2,500 cm^−1^ showed that the COOH functional group had been converted to another group. The chemical shifts of 2.923 and 2.985 ppm (single peak) in the ^1^H-NMR confirmed the existence of the CON(CH_3_)_2_ moiety. Next, the functional groups on the 3-position were modified in order to compare the relative antiproliferative activities of different groups. Compound **11** was converted to *N*,*N*-dimethyl-3-hydroxy-12-oxo-7-deoxycholic amide (**12**) by selective reduction using NaBH_4_. Finally, compound **13** was produced by the esterification of the 3-hydroxy group of **12**, and compound **14** was obtained by the reaction of **12** with thiosemicarbazide.

**Scheme 3:**
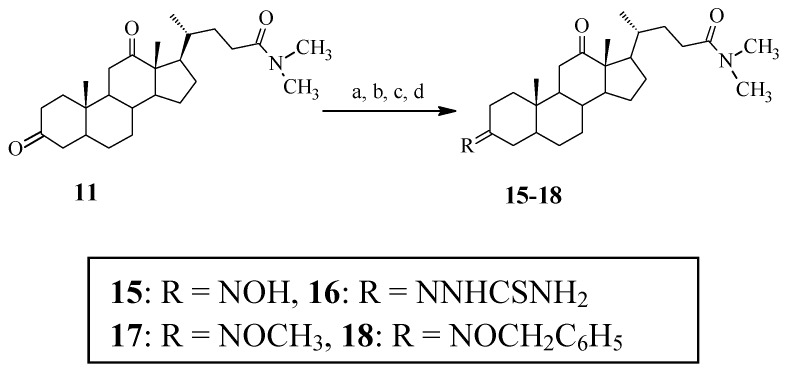
Synthetic procedures of *N*,*N*-dimethyl-7-deoxycholic amide derivatives.

To further determine the effect of the 3-substituent groups of *N*,*N*-dimethyl-7-deoxycholic amide derivatives on the cytotoxic activity, compounds **15**–**18** with different 3-substitutent groups were prepared. Similarly, the reaction of compound **11** with hydroxylamine hydrochloride, thiosemicarbazide, CH_3_ONH_2_·HCl or PhCH_2_ONH_2_·HCl afforded the corresponding products **15**, **16**, **17** or **18**, respectively (Scheme 3).

*Reagents and Conditions*: (a) NH_2_OH·HCl/95%EtOH, CH_3_COONa, 70 °C; (b) NH_2_NHCSNH_2_/EtOH, 80 °C; (c) CH_3_ONH_2_·HCl/95%EtOH, CH_3_COONa, 70 °C; (d) PhCH_2_ONH_2_·HCl/95% EtOH, CH_3_COONa, 70 °C.

### 2.2. *In Vitro* Evaluation of the Antiproliferative Activity

#### 2.2.1. Cell Culture and Assay for Cell Viability

SGC 7901 (human gastric carcinoma) and Bel 7404 (human liver carcinoma) cell lines were obtained from Guangxi Traditional Chinese Medical University. Cells were grown in RPMI-1640 supplemented with 10% cosmic calf serum (Hyclone, Beijing, China) and antibiotics in a humidified atmosphere of 5% CO_2_ at 37 °C. The viability of these cells was determined using the colorimetric CellTiter 96 aqueous Cell Proliferation Assay (MTT) according to the instructions provided by the manufacturer (Promega, Madison, WI, USA). Briefly, cells (1~3 × 10^4^ cells per well) were seeded in 96 wells plates. An equal amount of DMSO was added to the cells used as negative controls. All were treated in triplicate. One day after seeding, the cells were treated with or without different concentration of each compound and reincubated for 72 h. After the cells were washed with sterile phosphate buffer saline (PBS), 190 µL of RPMI-1640 and 10 µL of the tetrazolium dye (MTT) (5 mg/mL) solution were added to each well, and the cells were incubated for an additional 4 h. The medium was discarded; 200 µL of DMSO was added to dissolve the purple formazan crystals formed. The absorbance (A) at 492 nm was measured using a MULTISCAN MK3 analysis spectrometer (Thermo Scientific, Shanghai, China). The results were summarized as IC_50_ values in µ mol/L in Table 1.

**Table 1:** *In vitro* antiproliferative activities (IC_50_ in μmol/L) of the compounds **3**–**18**.

**Componuds**	**3**	**4**	**5**	**6**	**7**	**8**	**11**	**12**
SGC 7901	23.4	65.8	63.4	49.0	58.4	61.5	>100	>100
Bel 7404	51.2	>100	>100	80.1	>100	>100	>100	>100
Compounds	13	14	15	16	17	18	Cisplatin	
SGC 7901	>100	>100	>100	>100	>100	15.0	6.7	
Bel 7404	>100	>100	>100	>100	>100	14.4	23.2	

#### 2.2.2. Structure-Activity Relationships

As shown in Table 1, compound **3** with a 20-hydroximino group displayed distinct cytotoxicity against the two studied cancer cell lines. However, after the 20-hydroximino group of **3 ** was converted into an amide group (compounds **4**–**8**), the antiproliferative activity of compounds was remarkably decreased. The result could be ascribed to the blockage of the interaction of the amide group with outside groups, which may be a main reason causing a decrease of the cytotoxicity because of the steric hindrance of the steroidal nucleus [14]. Therefore, changing the hydroximino group to the amide group at position-20 did not increase the cytotoxic activities of these 17-acetamidoandrost-5-ene derivatives. Comparing the result with the earlier studies [12,13], we can see that the cytotoxicity of the steroidal amide compounds is not only dependent on the structure of the side chain, but also the position of the CONH functional group. When the compounds had a cholesteric side chain and A-ring or B-ring was modified into an A-lactam [13] or a B-lactam [19], it would display distinctive cytotoxicity against the cancer cells.

To investigate the effect of the 24-amide group on the biological function of the compounds, we synthesized some *N,N*-dimethyl-7-deoxycholic amide derivatives with various 3-substitutent groups (compounds **11**–**18**). Here, compounds **11**–**17** were almost inactive against the cancer cells. Once again, the reason may be the steric hindrance of *N,N*-dimethyl group inhibiting the affinity of the amide group with outside groups. Nevertheless, a 3-O-benzyloxime group played an important role in the cytotoxicity of compound **18** against these cancer cells. Compound **18** showed almost the same cytotoxicity as cisplatin to SGC 7901 and Bel 7404 cells (**18**: 15.0 and 14.4; cisplatin: 6.7 and 23.2 µmol/L), indicating that the presence of the 3-*O*-benzyloxime group on the compound **18** confers a positive effect on the cytotoxicity against these cancer cells, which provides clues the seeking more powerful anti-cancer drug candidates based on *O*-benzyloxime groups via structural optimization.

## 3. Experimental

Sterols and NaBH_4_ were purchased from Merck Co. (Shanghai, China) All chemicals and solvents were analytical grade and solvents were purified by general methods before being used. Melting points were determined on an X4 apparatus and were uncorrected. Infrared spectra were measured with a Thermo Scientific IS-10 Spectrophotometer. The ^1^H and ^13^C-NMR spectra were recorded in CDCl_3_ on a Bruker AV-300 spectrometer at working frequencies 300 and 75 MHz. Chemical shifts are expressed in parts per million (*δ*) values and coupling constants (*J*) in Hertz. LREIMS were recorded on a Thermo-DSQ instrument, while HREIMS were measured on a Agilent 6210 TOFMS instrument. 3-Acetoxy-17-acetamidoandrost-5-ene (**4**) and 3,12-dioxydeoxycholic acid (**10**) were prepared according to the literature [6] and [20], respectively.

*Synthesis of 3-hydroxy-17-acetamidoandrost-5-en* (**5**). K_2_CO_3_ solution (13%, 20 mL) was added to a solution of compound **4** (438 mg, 3.32 mmol) in CH_3_OH (200 mL). The reaction mixture was heated under reflux for 1 h. After completion of the reaction as indicated by TLC, the solvent was removed under vacuum. CH_2_Cl_2_ (200 mL) was added to dissolve the solid and the resulting solution was washed with cold water and saturated brine. After drying over anhydrous sodium sulfate, the solvent was removed under reduced pressure, and the resulting crude product was purified by chromatography on silica gel using methanol/dichloromethane (30:1) as eluent to give 709 mg (76%) of **5** as a white solid. *θ*_ mp_ 196–197 °C; IR (KBr) ν/cm^−1^: 3354, 2962, 2937, 1646, 1556, 1433, 1372, 1315; ^1^H-NMR (CDCl_3_, 300 MHz) *δ*: 0.721 (3H, s, 18-CH_3_), 1.028 (3H, s, 19-CH_3_), 2.002 (3H, s, N-COCH_3_), 2.36–2.24 (2H, m, C_4_-H), 3.58–3.51 (1H, m, C_3_-H), 3.908 (1H, q, *J* = 9.0, C_17_-H), 5.302 (1H, brd, *J* = 9.9, N-H), 5.367(1H, dd, *J* = 3.6, 1.8, C_6_-H); ^13^C-NMR (CDCl_3_, 75 MHz) *δ*: 169.9 (-NHCOCH_3_), 140.9 (5-C), 121.3 (6-C), 71.7 (3-C), 58.9 (17-C), 52.8 (9-C), 50.1 (13-C), 42.7 (4-C), 42.2 (14-C), 37.2 (10-C), 36.8(1-C), 36.7 (12-C), 32.0 (8-C), 31.6 (7-C), 31.5 (16-C), 28.7 (2-C), 27.7 (15-C), 23.6 (21-C), 20.6 (11-C), 19.4 (19-C), 11.9 (18-C); ESI-MS *m/z*: 332 (M+1)^+^.

*Synthesis of 17-acetamidoandrost-4-en-3,6-dione* (**6**). Jones’ reagent (0.7 mL, 0.267 mol/L) was added dropwise to the solution of **5** (709 mg) in acetone (30 mL) in 20 min. The mixture was stirred at room temperature for 1 h and then neutralized with 10% K_2_CO_3_ solution. The suspension was poured over a silica gel column and eluted with ethyl acetate. The solvent was removed under reduced pressure. The residue was purified by chromatography on silica gel using methanol/dichloromethane (20:1) as elution to give 590 mg (80%) of **6** as white solid. *θ*_ mp_ 198–199 °C; IR (KBr) ν/cm^−1^: 3321, 2941, 2864, 1691, 1654, 1556, 1442, 1384; ^1^H-NMR (CDCl_3_, 300 MHz) *δ*: 0.728 (3H, s, 18-CH_3_), 1.150 (3H, s, 19-CH_3_), 1.976 (3H, s, N-COCH_3_), 2.17–2.10 (2H, m, C_7_-βH and C_2_-αH), 2.495 (1H, dd, *J* = 13.8, 5.1, C_7_-αH), 2.662 (1H, dd, *J* = 15.9, 3.9, C_2_-βH), 3.937 (1H, q, *J* = 9.0, C_17_-H), 5.582 (1H, d, *J* = 9.0, N-H), 6.149 (1H, s, C_4_-H); ^13^C-NMR (CDCl_3_, 300 MHz) *δ*: 201.8 (6-C), 199.3 (3-C), 170.1 (-NHCOCH_3_), 160.7 (5-C), 125.6 (4-C), 58.5 (14-C), 52.7 (17-C), 50.9 (9-C), 46.3 (13-C), 43.0 (10-C), 39.7 (1-C), 36.3 (12-C), 35.5 (7-C), 34.3 (8-C), 33.9 (16-C), 28.2 (2-C), 23.4 (15-C), 23.2 (21-C), 20.5 (11-C), 17.6 (19-C), 11.9 (18-C); ESI-MS *m/z*: 344 (M+1)^+^.

*Synthesis of 3,6-dihydroxy-17-acetamidoandrost-4-en* (**7**). NaBH_4_ (42 mg, 1.1 mmol) was added at room temperature to a solution of **6** (124 mg, 0.36 mmol) in CH_3_OH (50 mL) in one portion. After 30 min, the reaction was stopped. The solution was neutralized with 1 M HCl. After evaporation of the majority of MeOH under reduced pressure, the residue was extracted with ethyl acetate (15 mL × 3). The organic layer was washed with cold water and saturated brines. After drying over anhydrous sodium sulfate, the solvent was removed under reduced pressure and a crude product was obtained. After recrystallization from methanol, 95 mg (78%) of compound **7** was obtained as a white solid. *θ*_ mp_ 198–200 °C; IR (KBr) ν/cm^−1^: 3331, 2941, 2866, 1654, 1442, 1384, 1217, 1131; ^1^H-NMR (CDCl_3_, 300 MHz) *δ*: 0.703 (3H, s, 18-CH_3_), 1.008 (3H, s, 19-CH_3_), 1.988 (3H, s, N-COCH_3_), 3.57–3.47 (1H, m, C_3_-H), 3.93–3.80 (1H, m, C_6_-H), 4.13 (1H, dd, *J* = 7.0, 1.5, C_17_-H), 5.36 (1H, d, *J* = 2.1, C_4_-H), 5.40 (1H, br s, N-H); ^13^C-NMR (CDCl_3_, 75 MHz) *δ*: 170.0 (-NHCOCH_3_), 140.9 (5-C), 121.3 (4-C), 71.6 (6-C), 68.8 (3-C), 58.9 (17-C), 52.8 (14-C), 50.1 (9-C), 42.7 (13-C), 42.2 (7-C), 37.2 (10-C), 36.8 (1-C), 36.5 (12-C), 32.1 (8-C), 31.6 (2-C), 31.5 (16-C), 28.7 (15-C), 23.6 (21-C), 20.6 (11-C), 19.4 (19-C), 12.0 (18-C); ESI-MS *m/z*: 348 (M+1)^+^. 

*Synthesis of 3-hydroxy-17-acetamidoandrost-4-en-6-one* (**8**). NaBH_4_ (42 mg, 1.1 mmol) was added to a solution of **6** (124 mg, 0.36 mmol) and NiCl_2_·6H_2_O (87 mg, 0.37 mmol) in CH_3_OH (15 mL) in the interval of 5 min at room temperature. After 5 min, the reaction was stopped. The solution was neutralized with 1 M HCl. After evaporation of the majority of MeOH under reduced pressure, water of 15 mL was added. Then the residue was extracted with ethyl acetate (15 × 3 mL). The resulting solution was washed with cold water and saturated brine. After drying over anhydrous sodium sulfate, the solvent was removed under reduced pressure, and the resulting crude product was purified by flash chromatography on silica gel using methanol/dichloromethane (20:1) as the eluent. Compound **8** was obtained as white solid (75 mg, 60%), *θ*_ mp_ 187–188 °C; IR(KBr) ν/cm^–1^: 3330, 2942, 2865, 1675, 1442, 1385, 1218; ^1^H-NMR (CDCl_3_, 300 MHz) *δ*: 0.747 (3H, s, 18-CH_3_), 1.196 (3H, s, 19-CH_3_), 2.004 (3H, s, N-COCH_3_), 2.22–2.14 (2H, m, C_16_-H), 2.418 (1H, dd, *J* = 12.9, 4.5, C_7_-αH), 3.929 (1H, q, *J* = 9.0, C_17_-H), 4.338 (1H, ddd, *J* = 11.4, 4.2, 1.2, C_3_-βH), 5.330 (1H, br d, *J* = 7.5, N-H), 6.196 (1H, d, *J* = 1.2, C_4_-H); ^13^C-NMR (CDCl_3_, 75 MHz) *δ*: 199.3 (6-C), 170.0 (-NHCO), 169.9 (5-C), 119.9 (4-C), 68.5 (3-C), 58.6 (17-C), 53.7 (9-C), 51.7 (14-C), 42.9 (13-C), 40.9 (7-C), 39.0 (10-C), 36.5 (8-C), 36.3 (1-C), 34.3 (12-C), 33.7 (2-C), 28.6 (16-C), 23.6 (15-C), 23.5 (21-C), 20.6 (11-C), 18.3 (19-C), 12.1 (18-C); ESI-MS *m/z*: 346 (M+1)^+^.

*Synthesis of N,N-dimethyl-3,12-dioxo-7-deoxycholic amide* (**11**). Oxalyl chloride (200 µL, 2.23 mmol) was added to a solution of **10** (535 mg, 1.38 mmol) and pyridine (150 µL, 1.80 mmol) in toluene (25 mL) at ice bath temperature. The mixture was stirred for 2 h. After completion of the reaction as indicated by TLC, a solution of dimethyl amine (540 µL) was added dropwise. The solution was stirred for 10 min at 0 °C and 10 mL ice water was added to the solution. The solution was extracted with dichloromethane (30 × 3 mL). The organic solution was washed with cold water and saturated brine. After drying over anhydrous sodium sulfate, the solvent was removed under reduced pressure, and the resulting crude product was purified by chromatography on silica gel using petroleum/ethyl acetate (1:2) as the eluent. Compound **11** was obtained as a white solid (480 mg, 71%), *θ*_ mp_ 271–272 °C; IR(KBr) ν/cm^–1^: 2929, 2859, 1695, 1634; ^1^H-NMR (CDCl_3_, 300 MHz) *δ*: 0.797 (3H, d, *J* = 5.7, 21-CH_3_), 1.000 (3H, s, 18-CH_3_), 1.050 (3H, s, 19-CH_3_), 2.13–2.04 (2H, m), 2.37–2.18 (3H, m), 2.58–2.48 (2H, m, C_11_-H), 2.872 (3H, s, NCH_3_), 2.955 (3H, s, NCH_3_); ^13^C-NMR (CDCl_3_, 75 MHz) *δ*: 214.2 (12-C), 212.1 (3-C), 173.4 (24-C), 58.5 (14-C), 57.5 (17-C), 46.5 (9-C), 44.2 (13-C), 43.6 (5-C), 42.1 (4-C), 38.3 (8-C), 37.3 (11-C), 36.8 (1-C), 36.7 (2-C), 35.6 (10-C), 35.5 (N-C), 35.4 (N-C), 35.3 (20-C), 30.6 (7-C), 30.2 (23-C), 27.4 (22-C), 26.5 (15-C), 25.4 (6-C), 24.3 (16-C), 22.1 (21-C), 18.7 (19-C), 11.7 (18-C); ESI-MS *m/z*: 416 (M+1)^+^.

*Synthesis of N,N-dimethyl-3-hydroxy-12-oxo-7-deoxycholic amide* (**12**). Compound **12** was prepared according to the procedure of **7**. Yield 30%, *θ*_ mp_ 279–280 °C; IR(KBr) ν/cm^–1^: 3465, 2925, 2859, 1707, 1621; ^1^H-NMR (CDCl_3_, 300 MHz) *δ*: 0.843 (3H, d, *J* = 6.3, 21-CH_3_), 0.986 (3H, s, 19-CH_3_), 0.995 (3H, s, 18-CH_3_), 2.26–2.17 (3H, m), 2.49-2.32 (2H, m, C_11_-H), 2.912 (3H, s, NCH_3_), 2.994 (3H, s, NCH_3_), 3.63-3.53 (1H, m, C_3_-H); ^13^C-NMR (CDCl_3_, 75 MHz) *δ*: 215.2 (12-C), 173.7 (24-C), 71.2 (3-C), 58.7 (14-C), 57.5 (17-C), 46.4 (13-C), 44.1 (5-C), 41.5 (9-C), 38.1 (8-C), 37.3 (11-C), 36.3 (1-C), 35.6 (4-C), 35.4 (10-C), 35.3 (N-C), 35.3 (N-C), 35.2 (20-C), 30.6 (2-C), 30.3 (23-C), 30.2 (22-C), 27.5 (6-C), 27.1 (15-C), 26.1 (16-C), 24.3 (7-C), 22.8 (21-C), 18.8 (18-C), 11.7 (19-C); ESI-MS *m/z*: 418 (M+1)^+^.

*Synthesis of N,N-dimethyl-3-acetoxy-12-oxo-7-deoxycholic amide* (**13**). Compound **13** was prepared by the esterification of **12** using acetic anhydride in pyridine. Yield: 78%. *θ*_ mp_ 153–154 °C; IR(KBr) ν/cm^–1^: 3440, 2953, 1730, 1700, 1656, 1465, 1447; ^1^H-NMR (CDCl_3_, 300 MHz)*δ*: 0.885 (3H, d, *J* = 6.3, 21-CH_3_), 1.035 (6H, br s, 18-CH_3_ and 19-CH_3_), 2.025 (3H, s, CH_3_CO-), 2.951 (3H, s, NCH_3_), 3.025 (3H, s, NCH_3_), 4.659–4.763 (1H, m, C_3_-H); ^13^C-NMR (CDCl_3_, 75 MHz) *δ*: 214.9 (12-C), 173.6 (24-C), 170.6 (CH_3_CO-), 73.7 (3-C), 58.7 (14-C), 57.6 (17-C), 46.5 (13-C), 44.1 (5-C), 41.4 (9-C), 38.1 (8-C). 37.3 (11-C), 35.7 (1-C), 35.4 (4-C), 35.3 (N-C), 34.9 (N-C), 32.1 (20-C), 30.7 (2-C), 30.3 (23-C), 27.5 (10-C), 26.9 (22-C), 26.4 (6-C), 26.4 (15-C), 24.4 (16-C), 22.7 (7-C), 21.4 (21-C), 18.8 (18-C), 11.7 (19-C); ESI-MS *m/z*: 460 (M+1)^+^.

*Synthesis of N,N-dimethyl-3-hydroxy-12-thiosemicarbazone-7-deoxycholic amide* (**14**). The compound **12** (100 mg, 0.24 mmol) was dissolved in 15 mL of CH_3_CH_2_OH and the solution was adjusted to pH ≈ 3–5 by adding a few drops of glacial acetic acid. After the solution was heated to 80 °C, and thiosemicarbazide (32 mg, 0.36 mmol) was added. The mixture was stirred for 10 h at 80 °C until no starting material (the progress of the reaction was monitored by TLC). Then the reaction was terminated and the majority of solvent was evaporated under reduced pressure. Water (10 mL) was added to the mixture which was then extracted with dichloromethane. The organic layer was washed with water and saturated brine. After drying over anhydrous sodium sulfate, the solvent was removed under reduced pressure, and the resulting crude product was purified by flash chromatography on silica gel using dichloromethane/methanol (40:1) as the eluent to give 56 mg of **14** as a white solid (yield: 49%), *θ*_ mp_ 294–295 °C; IR (KBr) *ν*/cm^–1^: 3427, 2921, 1634, 1503, 1401; ^1^H-NMR (CDCl_3_, 300 MHz): 0.843 (3H, d, *J* = 6.3, 21-CH_3_), 0.986 (3H, s, 18-CH_3_), 0.995 (3H, s, 19-CH_3_), 2.912 (3H, s, N-CH_3_), 2.994 (3H, s, N-CH_3_), 3.577 (1H, m, C_3_-H), 6.576 (1H, br s, -NH_2_), 7.234 (1H, br s, -NH_2_), 9.284 (1H, s, -NH-); ^13^C-NMR (CDCl_3_, 75 MHz): 178.8 (C=S), 173.6 (24-C), 162.6 (12-C), 71.1 (3-C), 58.7 (14-C), 57.5 (17-C), 46.4 (13-C), 44.1 (5-C), 41.6 (9-C), 38.1 (8-C), 37.4 (4-C), 36.3 (11-C), 35.7 (10-C), 35.6 (1-C), 35.4 (N-C), 35.3 (20-C), 35.1 (N-C), 30.7 (2-C), 30.3 (22-C), 30.2 (23-C), 27.5 (6-C), 27.1 (15-C), 26.1 (16-C), 24.4 (7-C), 22.8 (21-C), 18.8 (18-C), 11.7 (19-C); ESI-MS *m/z*: 491(M+1)^+^.

*Synthesis of N,N-dimethyl-3-hydroximino-12-oxo-7-deoxycholic amide* (**15**). Compound **11** (91 mg, 0.22 mmol) was dissolved in 95% CH_3_CH_2_OH (15 mL). After the mixture was heated to 70 °C, CH_3_COONa·3H_2_O (30 mg, 0.22 mmol) and NH_2_OH·HCl (18 mg, 0.26 mmol) were added. The mixture was stirred for 3 h at 70 °C. Then the reaction was terminated and the majority of solvent was evaporated under reduced pressure. Distilled water was added into the reaction mixture, and the product was extracted with dichloromethane. The combined extracts were washed with water and saturated brine, dried with anhydrous sodium sulfate, and evaporated under reduced pressure. The residue was purified by flash chromatography on silica gel using dichloromethane/methanol (40:1) as the eluent. Compound **15** was obtained as a white solid (56 mg, 60%). *θ*_mp_ 232–233 °C; IR (KBr) *ν*/cm^–1^: 3440, 1691, 1601, 1437, 1392; ^1^H-NMR (CDCl_3_, 300 MHz): 0.853 (3H, d, *J* = 6.3, 21-CH_3_), 1.025 (3H, s, 18-CH_3_), 1.035 (3H, s, 19-CH_3_), 2.534 (2H, m, C_11_-H), 2.923 (3H, s, N-CH_3_), 2.999 (3H, s, N-CH_3_), 3.070 (1H,dd, *J* = 14.4, 3.9, C_2_-*β*H); ^13^C-NMR (CDCl_3_, 75 MHz): 214.9 (12-C), 173.7 (24-C), 160.2 (3-C), 58.6 (14-C), 57.6 (17-C), 46.4 (9-C), 44.2 (5-C), 44.0 (13-C), 43.3 (10-C), 41.9 (8-C), 38.3 (11-C), 37.4 (N-C), 36.9 (N-C), 36.1 (20-C), 35.6 (7-C), 35.5 (4-C), 30.6 (23-C), 30.3 (6-C), 27.4 (22-C), 26.6 (2-C), 26.5 (1-C), 25.6 (15-C), 24.8 (16-C), 24.3 (21-C), 22.6 (19-C), 18.8 (18-C); ESI-MS *m/z*: 431(M+1)^+^.

*Synthesis of N,N-dimethyl-3-thiosemicarbazone-12-oxo-7-deoxycholic amide* (**16**). Compound **16** was prepared similarly to **14**, but from the compound **11**. Yield: 69%, *θ*_mp_ 282–283 °C. IR (KBr) *ν*/cm^–1^: 3432, 2929, 2868, 1703, 1638, 1499; ^1^H-NMR (CDCl_3_, 300 MHz): 0.856 (3H, d, *J* = 5.7, 21-CH_3_), 1.034 (3H, s, 18-CH_3_), 1.060 (3H, s, 19-CH_3_), 2.539 (2H, m, C_11_-H), 2.926 (3H, s, N-CH_3_), 3.002 (3H, s, N-CH_3_), 6.487 (1H, br s, N-H), 7.213 (H, br s, N-H), 8.873 (1H, s, -NH-); ^13^C-NMR (CDCl_3_, 75 MHz,): 214.5 (12-C), 178.7 (S=C), 173.6 (24-C), 156.8 (3-C), 58.5 (14-C), 57.5 (17-C), 46.5 (9-C), 44.4 (5-C), 44.1 (13-C), 43.7 (10-C), 42.3 (8-C), 38.3 (11-C), 37.3 (N-C), 36.1 (N-C), 36.0 (20-C), 35.6 (4-C), 35.4 (7-C), 30.6 (23-C), 30.3 (6-C), 29.9 (2-C), 27.4 (22-C), 26.7 (1-C), 25.6 (15-C), 24.3 (16-C), 22.5 (21-C), 18.8 (19-C), 11.8 (18-C); ESI-MS *m/z*: 489 (M+1)^+^. 

Compounds **17** and **18** were prepared similarly according to the procedure of **15**, but CH_3_ONH_2_·HCl and PhCH_2_ONH_2_·HCl were used as reagents instead of NH_2_OH·HCl.

*N,N-dimethyl-3-O-methyloximino-12-oxo-7-deoxycholic amide* (**17**). Yield: 70%, *θ*_mp_ 188–189 °C. IR (KBr) *ν*/cm^–1^: 3436, 2855, 1703, 1654, 1446, 1041; ^1^H-NMR (CDCl_3_, 300 MHz): 0.875 (3H, d, *J* = 6.3, 21-CH_3_), 1.043 (3H, s, 18-CH_3_), 1.054 (3H, s, 19-CH_3_), 2.55–2.40 (2H, m, C_11_-H), 2.940 (3H, s, N-CH_3_), 3.014 (3H, s, N-CH_3_), 3.804 (3H, s, O-CH_3_); ^13^C-NMR (CDCl_3_, 75MHz): 214.7 (12-C), 173.5 (24-C), 160.0 (3-C), 61.0 (-OCH_3_), 58.6 (14-C), 57.6 (17-C), 46.5 (9-C), 44.2 (13-C), 44.0 (10-C), 43.5 (5-C), 42.0 (8-C), 38.3 (11-C), 37.3 (N-C), 36.1 (N-C), 36.0 (20-C), 35.6 (4-C), 35.4 (7-C), 30.6 (23-C), 30.2 (6-C), 27.4 (22-C), 26.7 (2-C), 26.6 (1-C), 25.6 (15-C), 24.3 (16-C), 22.6 (21-C), 18.8 (19-C), 11.7 (18-C); ESI-MS *m/z*: 445 (M+1)^+^.

*N,N-dimethyl-3-O-benzyloximino-12-oxo-7-deoxycholic amide* (**18**). Yield: 72%, *θ*_mp_ 148–149 °C. IR (KBr) *ν*/cm^–1^: 2970, 2868, 1707, 1625, 1450, 1397; ^1^H-NMR (CDCl_3_, 300 MHz): 0.882 (3H, d, *J* = 6.3, 21-CH_3_), 1.048 (3H, s, 18-CH_3_), 1.056 (3H, s, 19-CH_3_), 2.35–2.45 (1H, m, C_11_-βH), 2.553 (1H, t, *J* = 12.6, C_11_-αH), 2.947 (3H, s, N-CH_3_), 3.019 (3H, s, N-CH_3_), 5.052 (2H, s, O-CH_2_-Ph), 7.36–7.29 (5H, m, -C_6_H_5_); ^13^C-NMR (CDCl_3_, 75 MHz): 214.7 (12-C), 173.5 (24-C), 160.5 (3-C), [138.2, 128.3, 128.3, 127.9, 127.8, 127.6 (-C6H5)], 75.2 (O-C), 58.6 (14-C), 57.5 (17-C), 46.5 (9-C), 44.3 (13-C), 44.0 (5-C), 43.5 (10-C), 41.9 (8-C), 38.3 (11-C), 37.3 (N-C), 36.1 (N-C), 36.0 (20-C), 35.6 (2-C), 35.4 (7-C), 30.6 (23-C), 30.3 (6-C), 27.4 (1-C), 26.6 (22-C), 25.8 (4-C), 25.6 (15-C), 24.3 (16-C), 22.6 (21-C), 18.8 (19-C), 11.8 (18-C); ESI-MS *m/z*: 521 (M+1)^+^.

## 4. Conclusions

We have prepared some 17-acetamidoandrostane and *N,N*-dimethyl-7-deoxycholic amide derivatives with different substitutent groups on different positions of the steroidal nucleus. The antiproliferative activity of the synthesized compounds against the SGC 7901 and Bel 7404 cell lines was assayed. The results showed that the blockage of the interaction of the amide group with outside groups might cause a decrease of the cytotoxicity, and an *O*-benzyloxime group on the 3-position of *N,N*-dimethyl-7-deoxycholic amide could enhance the cytotoxic activity of the compound. Our result revealed some structure-activity relationships of the compounds and although some displayed indistinctive cytotoxicity against these cancer cells, this informstion may be useful for the design of novel chemotherapeutic drugs with better activity.
